# For the Benefit of the Unfortunate Young Man

**Published:** 1897-03

**Authors:** F. B. McRae, J. W. Cole, Benj. Frenkel, W. H. Lancaster

**Affiliations:** Temple, Texas; Kennedale, Texas; Graduate of University of Texas, Medical Deparment; Galveston, Texas; Moulton, Texas


### Correspondence.

### For the Benefit of the Unfortunate Young Man.

TEMPLE, TEXAS, Feb. 15, 1897.

*Editors Texas Medical Journal:*

In response to your appeal in behalf of an "Unfortunate Young Man," I offer the following, with a hope that it may be of direct good, or indirect, in suggesting thoughts upon which sone one else may further build to the desired end.

First. We have to contend with an irregularly developed system.

Second. We have the semi-developed organs, continually imposed upon by the stronger and fuller developed organs.

Third. We have a typically well marked train of reflex neuroses.

In the rational treatment of any *perverted vascular action* of a system, we should first find the cause, and in the treatment, we make the first step in removing that cause; to that end, a thorough systematic physical examination is essentially the first step; and allowing ourselves to presume, for the sake of presenting our ideas, we infer that the patient under discussion suffers with an *elongated* and *adherent prepuce*, which has largely prevented a healthy amount of blood necessary for the proper development and nourishment of the organ.

When the circulation is interfered with in any organ, we have more or less pain and discomfort, with more or less nervous derangement, which leads, often, to more disturbing influences, and soon, reaching, either *atrophy* or *engorgement;* and that, with the rubbing and chafing by the clothes, as in this type of cases, is usually the step-by-step, that leads up to to the act of masturbation which, when once started, needs little to have the act repeated, with that rapidity which makes such inroads into the system, before recognized.

When one organ becomes more or less behind in the general development of the system it is easy for it to be imposed upon, and ever afterward unable to keep even pace in growth with its fellows, and discharge its functions, and too often, not only never catches up, but its growth is entirely arrested, and we have so often the so-called infantile type of an organ.

As the general system has its motor and sensory nerve-supply so perfectly throughout the system that any locality's nerves of feeling becoming affected we have the effect transmitted unerringly to the *nerve centres* and reflected back through the motor system often to other and weaker organs, and with a special tendency toward those of locomotion.

To sum up the conditions expected to be met in this class of eases, we have a general anæmic condition, with deranged nervous system, poor appetite, impaired digestion, inefficient peristalic action of the bowels, and consequently constipation, often to an alarming degree, with a general feeling of nervous discomfort, and the noticeable desire for seclusion.

The prognosis depends largely upon the age of the patient and the extent of the self-abuse, but is usually favorable if the patient can be induced to persist in treatment and carry out the direction of his advisor.

Treatment must be arranged for each individual case, and no set rule can be laid down to meet the different types and stages of this trouble. Environments, age and general development, with individual idiosyncrasies should modify the treatment of all cases. The secretory and excretory organs must have such attention as is needed, while the general system should have tonics. Massage to the extremities regularly and consistently to advisable. If there is an elongated prepuce, whether adherent or not, it should be removed. If there is a stricture of the urethra it should be cut or dilated. Regular out-door exercise, together with a nourishing diet, largely farinaceous, with small amount of beef, fish, or fowls at intervals as deemed best. Coffee, tea, sweet milk, buttermilk or cocoa are admissible. *Sexual intercourse* will add nothing to the treatment for a typical *disease.* I would not advise *marriage.*

This hurriedly sketched off plan I have adopted in a number of similar cases, and it has proved efficient, and pleasing in results. I give it to the readers of the "Red Back" for what it may be worth to "the unfortunate young man."

Very truly,

F. B. MacRae, M. D.

---

### That Young Man.

Kennedale, Texas, Feb. 15, 1897.

*Editors Texas Medical Journal:*

Through sympathy for the unkown young man who so pathetically pleads for medical advice, I will report a case that I think will be of interest to him, and may possibly interest some of the medical fraternity:

A young man, Mr. S——, aged 20, studying medicine under me, became so anæmic and emaciated, I, while driving along the road one day, got to talking to him in a confidential manner, and I asked him why he seemed so dull and listless, and what caused him to be forever in a profound study, seeming to have no life and to take no interest in anything.

I told him I had been noticing him closely for some time, and had been watching his decline, and had thought of writing his father and advise him to take him out home in the country. To this he objected. And I said to him, "John, you are under my care, and I feel an interest in you, and I want you to trust me and consider me your friend." At this he broke down, and told me one of the most pitiable confessions I ever listened to. He told me that he had been addicted to masturbation for four years and that he could not quit it. He said he would wake himself up at night performing this awful job, and also stated that he had nightly emissions. He would perform this operation two or three times a day and then have emissions in the night. This young man was truly in an awful sad condition, and I was very much puzzled to decide what to do with him.

To going home he objected, and promised me, if I would treat him, and not let his father know his condition, he would do anything I asked him to do. He had almost dispaired of living, but could not pick up courage to commit suicide. I finally, reluctantly, agreed to do as he wished, with the distinct understanding that he must follow my directions. He stayed in my office day and night, and had it filled with novels of the dime sensational order, cheap literature. I at once went to work and picked up everything of the kind in the room and put it in the stove and burned them up, and made him promise not to buy, borrow nor read anything more on that line. This done, I made him strip, and examined him from head to foot and found everything normal, except, I think, he had the longest prepuce I ever saw, and under this he allowed filth to accumulate. I told him that this would have to come off, which he agreed to after considerable whining. So on the third day after I took him in charge, I performed this little operation which, I think, did more than any one thing to accomplish his complete cure. I found this young man suffered from constipation which, I think, was caused by the self-abuse. I gave him a prescription for aro. syrup of rhu., fluid ext. cas. sag., equal parts, which caused his bowels to move once or twice a day. He took two teaspoonfuls at bed time, and would repeat in the morning when necessary. I also gave him strychnine, $\frac{1}{30}$ of a grain, four times daily. I also gave a prescription for bromide pot. tr. valerian and chloral hydrate. In the beginning I had him to take a dose of the last named mixture, about every four hours. In connection with this I had him to take a bath every day, and in the office I had him to keep a tub of water, and whenever he felt any desire for his old habit—just as soon as he felt any signs of an erection—I would have him drop his pants and sit down in this tub of cold water, for it was in December I treated him. He said that was the toughest part of the treatment, but he said it cooled things off, and I guess it did, for I have seen the broken ice in the tub where he had been using it. I advised him to stay away from women altogether, or as near so as possible, and keep his mind occupied. Now, this is the case of one young man over whom I had almost hourly oversight, and I know he is cured, and is now practicing medicine in East Texas, and I expect he will read this piece when it comes out. He is doing a nice practice, has a wife, and one of the sweetest little girls I ever saw; and the above is all I did for him, and I believe it will do the same for this young man. I would advise him to place himself under the care of some physician, and if he has a prepuce have it cut off the first thing, and keep out of female society, and refrain from reading light literature, and use some will power, and he will come out all O. K. I seldom write for the press, nor would I now, but for the sympathy I feel for this young fellow who is ruined unless he brings a halt.

J. W. COLE, M. D.

---

### The Unfortunate Young Man.

AUSTIN, TEXAS, February 13, 1897.

*Editor of the Texas Medical Journal:*

In reply to your recent request that a line of treatment be suggested for "an unfortunate young man," who is a victim—of several years's standing—to the habit of masturbation, I will say that without a definite knowledge as to the condition of his prostatic urethra, no rational method of treatment can be offerred. In fully 90 per cent of the confirmed masturbators hyperæsthesia of the prostatic urethra exists; in many, a granular condition of the Verumontanum, or a subacute or chronic inflammation of the urethra proper, giving rise to exalted and perverted sexual impulses. A cure of the habit and the accompanying spermatorrhœa, or impotence, which eventually follows, necessarily depends upon the cure of this local condition; this requires topical applications. In these cases the too often routine, and early miscellaneous administration of the aphrodisiacs (strychnia, damiania, etc.), and the cold sitz bath, is like adding fuel to the fire, and is irrational, to say the least. The sedative plan of treatment is a step in the right direction, but with it, alone, we can never hope to perfect a cure, and are only temporizing with measures. Every case is a law unto itself. The existence of a myelasthenia, or of variocecle, phymosis, inflammation of the seminal vesicles or sympexions of their ducts, and numerous other recognized sources of reflex irritation, must be examined into, and eliminated, before we are in a position to treat scientifically and successfully this formidable disease.

As to the existence of atrophy of the genitals: One who has had much experience with this class of patients knows that the most common and persistent delusion with sexual hypochrondriacs is this idea of shrinking of their privates, and doubtless this young man is a case in point. There can be but one advice to give him. Let him seek some honest and reputable physician who has given the subject and the treatment of the male sexual organs some attention.

J. S. W., M. D.

---

### Spider Bite.

KENNEY, TEXAS, Feb. 14, 1897.

*Editors Texas Medical Journal:*

### The Unfortunate Young Man.

GALVESTON, TEXAS, February 11, 1897.

*Editor Texas Medical Journal:*

DEAR SIR: Noticing in your JOURNAL, edition of this month, a paragraph entitled "Help Wanted," under the head of "Correspondence," I take great pleasure in volunteering to alleviate this unfortunate person of his loathsome affliction.

In order that the poor unfortunate may receive every attention possible, would suggest that he come to Galveston and receive a permit to enter the Sealy Hospital, or St. Mary's Infirmary, where I will treat him gratuitously by hypnotic therapeusis; and which, to my mind, is the only remedial and positive cure for mental and nervous diseases. You can inform this poor individual of my offer, and assure him that he will be greatly benefitted, if not entirely cured, of his malady. Hoping this to be satisfactory to both parties,

I am yours fraternally,

BENJ. FRENKEL, M. D.,
Graduate of University of Texas, Medical Deparment.

---

### Still Another Help.

MOULTON, TEXAS, February 23, 1897.

*Editors Texas Medical Journal:*

With the hope of proving beneficial to "An Unfortunate Young Man," I make the following suggestions and outlines of treatment:

We advise first, quit the practice. This is absolute, and should be both mechanically and mentally. What will assist the young man to give up his unhealthful (really diseased condition) practice? First, if possible, obtain constant, satisfactory, and, if possible, remunerative occupation or employment, not necessarily hard labor, but such as will keep him constantly busy, and which of itself attaches some responsibility. This were better in outdoor atmosphere.

The most essential point in reference to this occupation is that it be such as will place the party in constant contact with refined, intellectual, congenial company or associates. If possible, keep good company both day and night. Have a bed companion, if it must be, a male. The point is to place such restraints around the victim as will force absolute abstenanace, and prove an assistance to the will power—a preoccupancy of the mind. I object not if the company be of the opposite sex, if of the proper kind. It is ennobling, refining, encouraging, civilizing. "Healthful mental occupation and chaste associations do very much toward effecting a cure."

To carry out these objects of treatment, as well as the minor (seemingly minor) details, requires an exercise of will power, perseverence and determination, of which, we hope, the young man is still possessed, from the fact that he makes such an earnest appeal for help. This very desire for relief, and the interest he manifests in himself, together with the pride still evinced in the desire to be a man again, makes strong our hopes to see this young man restored to health and usefullness.

Second. Good nourishment is an essential. He should have such food as satisfies the appetite and builds tissues, and such as does not tax too heavily the digestive apparatus. Really, constructives. This we can scarcely outline, for no two people are just alike.

Third. Indulge in a cold shower bath every morning (before dressing is better). It may prove quite a task, a hard and cold one, at first, but it will aid very materially if persisted in, and if continued will in future years prove a pleasant and indispensable essential to comfort, health, a general good feeling, and an adjunct to longevity. Some application to books or literature is beneficial. Let him get interested in the topics and issues of the day; keep up with them, and not give himself over to individualism. Too much or too constant exercise on horseback sometimes stimulates, or irritates, the sexual appendages, and probably the centers, too. It were better to avoid this, especially when alone. Let him sleep on a hard bed always, and under light cover. A resort to a suitable sanitarium may sometimes become necessary, but rather, if possible, let him make his own sanitarium. Have a large one, the entire county or great State. I don't like nerve sedatives, anaphrodisiacs. This is not a condition of over health, but one of denutrition. As for medical treatment, I suggest, as a nerve tonic and constructive, the dypsomania tablet, so oft prescribed, as good for this condition, which is as follows:

℞ Gold and sodii chlor............gr. 1-24
Strych. nit..........................gr. 1-40
Glonoin..............................gr. 1-100
Atropine.............................gr. 1-200
Tr. digitalis.........................M. 3
Oleoresin capsicum ..............gr. $\frac{1}{8}$
M. Ft. tablets, one to be taken just before meals.

As a tonic, laxative and digestive, we suggest the following to be taken, one tablet one hour after each meal. Both these preparations to be used quite a while.

No. 2 ℞ is as follows:

Ox gall inspissated..................gr. i.
Pure pancreatine.....................gr. i.
Pure pepsin...........................gr. i.
Ext. colocynth........................gr. $\frac{1}{4}$
Quine. hydrochlor..................gr. i.
Ext. nucis vom......................gr. 1-12
Ext. tarraxacum....................gr. $\frac{1}{2}$
Pulv. ferri............................gr. i.
M. Ft. tablet.......................j.
Sig: To be taken one hour after each meal.

If there exist a pale atonic condition of urethra, we believe the occasional introduction of the cold steel sound does good—yet not often needed.

We hope our suggestions are not too profuse, for we believe if the young man will persist in the use of these remedies, and every suggestion herein made, the reward will be, a man restored to health, and confidence re-established in his own respect and that of his friends.

Respectfully,

W. H. LANCASTER, M. D.

